# (*E*)-3-(2*H*-1,3-Benzodioxol-5-yl)-1-(7-hy­droxy-5-meth­oxy-2,2-dimethyl­chroman-8-yl)prop-2-en-1-one

**DOI:** 10.1107/S1600536811031321

**Published:** 2011-08-11

**Authors:** Farediah Ahmad, Nur Athirah Hashim, Norazah Basar, Khalijah Awang, Seik Weng Ng

**Affiliations:** a Department of Chemistry, Universiti Teknologi Malaysia, 81310 Skudai, Johor, Malaysia; b Department of Chemistry, University of Malaya, 50603 Kuala Lumpur, Malaysia; c Chemistry Department, Faculty of Science, King Abdulaziz University, PO Box 80203 Jeddah, Saudi Arabia

## Abstract

The reaction of 5,6-(2,2-dimethyl­chromane)-2-hy­droxy-4-meth­oxy­acetophenone and 3,4-methlene­dioxy­benzaldehyde affords the title chalcone derivative, C_22_H_22_O_6_. The two benzene rings are connected through a —C(=O)—CH=CH— (propenone) unit, which is in an *E* conformation; the ring with the hy­droxy substitutent is aligned at 6.2 (1)° with respect to this unit, whereas the ring with the methyl­enedi­oxy substituent is aligned at 8.2 (1)°. The dihdral angle between the rings is 14.32 (7)°. The hy­droxy group engages in an intra­molecular hydrogen bond with the carbonyl O atom of the propenone unit, generating an *S*(5) ring.

## Related literature

For a related structure and background to chalcones, see: Hashim *et al.* (2011 ▶).
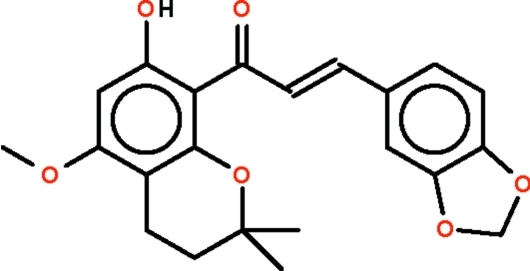


## Experimental

### 

#### Crystal data

C_22_H_22_O_6_
*M* *_r_* = 382.40
Triclinic, 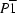

*a* = 9.4531 (7) Å
*b* = 10.4462 (8) Å
*c* = 10.8426 (8) Å
α = 113.866 (1)°
β = 90.120 (1)°
γ = 109.882 (1)°
*V* = 908.55 (12) Å^3^
*Z* = 2
Mo *K*α radiation
μ = 0.10 mm^−1^
*T* = 100 K
0.35 × 0.25 × 0.05 mm

#### Data collection

Bruker SMART APEX CCD diffractometer
8722 measured reflections
4141 independent reflections
3554 reflections with *I* > 2σ(*I*)
*R*_int_ = 0.028

#### Refinement

*R*[*F* ^2^ > 2σ(*F* ^2^)] = 0.043
*wR*(*F* ^2^) = 0.126
*S* = 1.03
4141 reflections
257 parameters
1 restraint
H atoms treated by a mixture of independent and constrained refinement
Δρ_max_ = 0.33 e Å^−3^
Δρ_min_ = −0.34 e Å^−3^

### 

Data collection: *APEX2* (Bruker, 2009 ▶); cell refinement: *SAINT* (Bruker, 2009 ▶); data reduction: *SAINT*; program(s) used to solve structure: *SHELXS97* (Sheldrick, 2008 ▶); program(s) used to refine structure: *SHELXL97* (Sheldrick, 2008 ▶); molecular graphics: *X-SEED* (Barbour, 2001 ▶); software used to prepare material for publication: *publCIF* (Westrip, 2010 ▶).

## Supplementary Material

Crystal structure: contains datablock(s) global, I. DOI: 10.1107/S1600536811031321/hb6346sup1.cif

Structure factors: contains datablock(s) I. DOI: 10.1107/S1600536811031321/hb6346Isup2.hkl

Supplementary material file. DOI: 10.1107/S1600536811031321/hb6346Isup3.cml

Additional supplementary materials:  crystallographic information; 3D view; checkCIF report

## Figures and Tables

**Table 1:** Hydrogen-bond geometry (Å, °)

*D*—H⋯*A*	*D*—H	H⋯*A*	*D*⋯*A*	*D*—H⋯*A*
O3—H3⋯O4	0.86 (1)	1.63 (1)	2.453 (1)	158 (2)

**Fig. 1.**
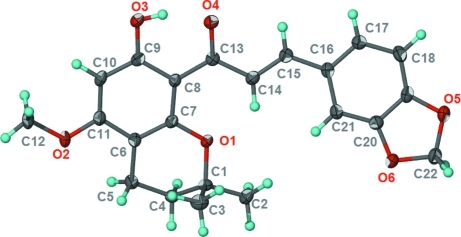
The molecular structure of (I) at the 70% probability level; hydrogen atoms are drawn as spheres of arbitrary radius.

## supplementary crystallographic information

### Comment

We intend to use the intensely yellow-orange title compound, (I), in the synthesis of other compounds. A related structure was reported in the previous paper. Its two benzene rings are connected through the –C(═ O)–CH═CH– unit, which is of an *E* configuration; the ring with the hydroxy substitutent is aligned at 6.2 (1) ° with this unit whereas the ring with the methyleledioxy substituent is aligned at 8.2 (1) °. The hydroxy group engages in intramolecular hydrogen bonding with the carbonyl O atom of the unit (Fig. 1).

### Experimental

A solution of 2-hydroxy-4-methoxy-5,6-(2,2-dimethylchromane)acetophenone (150 mg, 0.68 mmol) and methylenedioxybenzaldehyde (150 mg, 0.45 mmol) in ethanol (10 ml) was treated with 50% potassium hydroxide (1 ml). The mixture was stirred for 48 h. The mixture was poured into iced water (30 ml); this was acidified with 10% hydrochloric acid. The mixture was extracted with dichloromethane (3 *x* 20 ml). The organic layer was washed with water (3 *x* 10 ml) and brine (3 *x* 5 ml) followed by drying over anhydrous magnesium sulfate. The solvent was evaporated under reduced pressure to yield a dark orange syrup. The syrup was subjected to VLC for purification by using silica gel and eluting with a hexane:ethyl acetate solvent system (9:1) to give (I) (520 mg, 30%) as yelloiwsh orange blocks, m.p. 395–399 K. The formulation was established by ^1^H– and ^13^C-NMR spectroscopy.

### Refinement

Carbon-bound H-atoms were placed in calculated positions (C—H 0.95 to 0.98 Å) and were included in the refinement in the riding model approximation, with *U*(H) set to 1.2 to 1.5*U*_eq_(C).

The hydroxy H-atom was located in a difference Fourier map, and was refined with a distance restraint of O–H 0.84±0.01 Å; its temperature factor was freely refined.

Omitted from the refinement were (-3 3 - 8), (-2 8 - 1), (1 1 - 4) (-4 9 3) and (-3 0 16).

### Crystal data

**Table:**

C_22_H_22_O_6_	*Z* = 2
*M**_r_* = 382.40	*F*(000) = 404
Triclinic, *P*1	*D*_x_ = 1.398 Mg m^−^^3^
Hall symbol: -P 1	Mo *K*α radiation, λ = 0.71073 Å
*a* = 9.4531 (7) Å	Cell parameters from 3990 reflections
*b* = 10.4462 (8) Å	θ = 2.3–28.3°
*c* = 10.8426 (8) Å	µ = 0.10 mm^−^^1^
α = 113.866 (1)°	*T* = 100 K
β = 90.120 (1)°	Block, yellow orange
γ = 109.882 (1)°	0.35 × 0.25 × 0.05 mm
*V* = 908.55 (12) Å^3^	

### Data collection

**Table:**

Bruker SMART APEX CCD diffractometer	3554 reflections with *I* > 2σ(*I*)
Radiation source: fine-focus sealed tube	*R*_int_ = 0.028
graphite	θ_max_ = 27.5°, θ_min_ = 2.3°
ω scans	*h* = −12→12
8722 measured reflections	*k* = −13→13
4141 independent reflections	*l* = −12→14

### Refinement

**Table:**

Refinement on *F*^2^	Primary atom site location: structure-invariant direct methods
Least-squares matrix: full	Secondary atom site location: difference Fourier map
*R*[*F*^2^ > 2σ(*F*^2^)] = 0.043	Hydrogen site location: inferred from neighbouring sites
*wR*(*F*^2^) = 0.126	H atoms treated by a mixture of independent and constrained refinement
*S* = 1.03	*w* = 1/[σ^2^(*F*_o_^2^) + (0.0704*P*)^2^ + 0.3083*P*] where *P* = (*F*_o_^2^ + 2*F*_c_^2^)/3
4141 reflections	(Δ/σ)_max_ = 0.001
257 parameters	Δρ_max_ = 0.33 e Å^−^^3^
1 restraint	Δρ_min_ = −0.34 e Å^−^^3^

### Special details

**Table:**

Geometry. All e.s.d.'s (except the e.s.d. in the dihedral angle between two l.s. planes) are estimated using the full covariance matrix. The cell e.s.d.'s are taken into account individually in the estimation of e.s.d.'s in distances, angles and torsion angles; correlations between e.s.d.'s in cell parameters are only used when they are defined by crystal symmetry. An approximate (isotropic) treatment of cell e.s.d.'s is used for estimating e.s.d.'s involving l.s. planes.

### Fractional atomic coordinates and isotropic or equivalent isotropic displacement parameters (Å^2^)

**Table:**

	*x*	*y*	*z*	*U*_iso_*/*U*_eq_	
O1	0.24900 (10)	0.26722 (10)	0.37397 (9)	0.0175 (2)	
O2	0.14558 (11)	0.35255 (11)	0.81933 (9)	0.0212 (2)	
O3	0.65288 (11)	0.43380 (11)	0.73541 (10)	0.0205 (2)	
H3	0.695 (2)	0.423 (2)	0.6631 (14)	0.041 (5)*	
O4	0.70562 (11)	0.37601 (11)	0.50151 (10)	0.0202 (2)	
O5	0.54846 (12)	0.07418 (12)	−0.34009 (10)	0.0248 (2)	
O6	0.33410 (11)	0.02763 (12)	−0.23687 (9)	0.0236 (2)	
C1	0.08521 (14)	0.19138 (15)	0.32290 (13)	0.0173 (3)	
C2	0.06859 (17)	0.19318 (18)	0.18445 (14)	0.0246 (3)	
H2A	0.0903	0.2968	0.1971	0.037*	
H2B	−0.0358	0.1293	0.1361	0.037*	
H2C	0.1405	0.1547	0.1308	0.037*	
C3	0.03668 (17)	0.03067 (15)	0.30829 (15)	0.0238 (3)	
H3A	0.0422	0.0322	0.3992	0.036*	
H3B	0.1048	−0.0163	0.2569	0.036*	
H3C	−0.0682	−0.0274	0.2594	0.036*	
C4	0.00435 (15)	0.28290 (15)	0.42117 (13)	0.0169 (3)	
H4A	−0.1065	0.2362	0.3862	0.020*	
H4B	0.0423	0.3864	0.4273	0.020*	
C5	0.03195 (15)	0.28986 (16)	0.56239 (13)	0.0193 (3)	
H5A	0.0064	0.3728	0.6305	0.023*	
H5B	−0.0353	0.1941	0.5633	0.023*	
C6	0.19652 (15)	0.31572 (14)	0.60088 (13)	0.0166 (3)	
C7	0.29656 (15)	0.30719 (14)	0.50733 (13)	0.0150 (3)	
C8	0.45543 (14)	0.34365 (14)	0.54542 (12)	0.0147 (3)	
C9	0.50582 (15)	0.39096 (14)	0.68644 (13)	0.0165 (3)	
C10	0.40485 (16)	0.39162 (15)	0.77956 (13)	0.0181 (3)	
H10	0.4402	0.4177	0.8718	0.022*	
C11	0.25254 (15)	0.35386 (14)	0.73640 (13)	0.0171 (3)	
C12	0.19315 (18)	0.38194 (19)	0.95672 (14)	0.0267 (3)	
H12A	0.1055	0.3751	1.0053	0.040*	
H12B	0.2718	0.4834	1.0033	0.040*	
H12C	0.2347	0.3074	0.9556	0.040*	
C13	0.56815 (15)	0.33381 (14)	0.45276 (13)	0.0163 (3)	
C14	0.52921 (15)	0.27293 (15)	0.30357 (13)	0.0185 (3)	
H14	0.4253	0.2236	0.2613	0.022*	
C15	0.63933 (15)	0.28671 (14)	0.22708 (13)	0.0171 (3)	
H15	0.7415	0.3368	0.2738	0.021*	
C16	0.61775 (15)	0.23228 (14)	0.07876 (13)	0.0169 (3)	
C17	0.74549 (16)	0.25991 (15)	0.01505 (14)	0.0198 (3)	
H17	0.8436	0.3131	0.0699	0.024*	
C18	0.73502 (16)	0.21236 (16)	−0.12671 (14)	0.0216 (3)	
H18	0.8229	0.2326	−0.1688	0.026*	
C19	0.59114 (16)	0.13521 (15)	−0.20110 (13)	0.0189 (3)	
C20	0.46250 (15)	0.10673 (14)	−0.13959 (14)	0.0179 (3)	
C21	0.47095 (15)	0.15306 (15)	−0.00160 (13)	0.0185 (3)	
H21	0.3817	0.1329	0.0388	0.022*	
C22	0.38461 (16)	0.01443 (15)	−0.36479 (14)	0.0215 (3)	
H22A	0.3466	0.0718	−0.4011	0.026*	
H22B	0.3454	−0.0927	−0.4325	0.026*	

### Atomic displacement parameters (Å^2^)

**Table:**

	*U*^11^	*U*^22^	*U*^33^	*U*^12^	*U*^13^	*U*^23^
O1	0.0143 (5)	0.0245 (5)	0.0121 (4)	0.0059 (4)	0.0001 (3)	0.0074 (4)
O2	0.0229 (5)	0.0284 (5)	0.0128 (4)	0.0087 (4)	0.0059 (4)	0.0100 (4)
O3	0.0173 (5)	0.0277 (5)	0.0145 (5)	0.0067 (4)	−0.0005 (4)	0.0088 (4)
O4	0.0163 (5)	0.0251 (5)	0.0179 (5)	0.0075 (4)	0.0015 (4)	0.0081 (4)
O5	0.0244 (5)	0.0304 (5)	0.0141 (5)	0.0068 (4)	0.0046 (4)	0.0071 (4)
O6	0.0191 (5)	0.0315 (5)	0.0141 (5)	0.0048 (4)	0.0009 (4)	0.0078 (4)
C1	0.0131 (6)	0.0203 (6)	0.0164 (6)	0.0044 (5)	0.0003 (5)	0.0073 (5)
C2	0.0212 (7)	0.0356 (8)	0.0157 (6)	0.0103 (6)	0.0000 (5)	0.0102 (6)
C3	0.0218 (7)	0.0203 (7)	0.0268 (7)	0.0081 (6)	0.0020 (6)	0.0077 (6)
C4	0.0140 (6)	0.0199 (6)	0.0176 (6)	0.0060 (5)	0.0016 (5)	0.0091 (5)
C5	0.0172 (6)	0.0244 (7)	0.0173 (6)	0.0084 (5)	0.0044 (5)	0.0095 (5)
C6	0.0177 (6)	0.0177 (6)	0.0147 (6)	0.0062 (5)	0.0031 (5)	0.0076 (5)
C7	0.0170 (6)	0.0145 (6)	0.0135 (6)	0.0053 (5)	0.0007 (5)	0.0065 (5)
C8	0.0161 (6)	0.0152 (6)	0.0127 (6)	0.0054 (5)	0.0015 (5)	0.0062 (5)
C9	0.0182 (6)	0.0143 (6)	0.0153 (6)	0.0049 (5)	−0.0002 (5)	0.0058 (5)
C10	0.0226 (7)	0.0196 (6)	0.0120 (6)	0.0073 (5)	0.0007 (5)	0.0072 (5)
C11	0.0202 (7)	0.0165 (6)	0.0140 (6)	0.0058 (5)	0.0035 (5)	0.0070 (5)
C12	0.0299 (8)	0.0412 (9)	0.0154 (7)	0.0162 (7)	0.0080 (6)	0.0158 (6)
C13	0.0179 (6)	0.0148 (6)	0.0165 (6)	0.0064 (5)	0.0023 (5)	0.0067 (5)
C14	0.0175 (6)	0.0206 (6)	0.0155 (6)	0.0067 (5)	0.0008 (5)	0.0063 (5)
C15	0.0182 (6)	0.0174 (6)	0.0166 (6)	0.0078 (5)	0.0024 (5)	0.0072 (5)
C16	0.0200 (7)	0.0172 (6)	0.0159 (6)	0.0088 (5)	0.0049 (5)	0.0077 (5)
C17	0.0176 (6)	0.0220 (6)	0.0190 (7)	0.0074 (5)	0.0027 (5)	0.0082 (5)
C18	0.0209 (7)	0.0247 (7)	0.0202 (7)	0.0097 (6)	0.0084 (5)	0.0097 (5)
C19	0.0243 (7)	0.0184 (6)	0.0139 (6)	0.0093 (5)	0.0044 (5)	0.0060 (5)
C20	0.0180 (6)	0.0169 (6)	0.0179 (6)	0.0062 (5)	0.0024 (5)	0.0070 (5)
C21	0.0186 (7)	0.0214 (6)	0.0163 (6)	0.0080 (5)	0.0051 (5)	0.0085 (5)
C22	0.0248 (7)	0.0209 (6)	0.0152 (6)	0.0067 (6)	0.0035 (5)	0.0059 (5)

### Geometric parameters (Å, °)

**Table:**

O1—C7	1.3595 (15)	C6—C11	1.4063 (18)
O1—C1	1.4625 (15)	C7—C8	1.4325 (18)
O2—C11	1.3548 (16)	C8—C9	1.4262 (17)
O2—C12	1.4314 (16)	C8—C13	1.4666 (18)
O3—C9	1.3402 (16)	C9—C10	1.3905 (18)
O3—H3	0.862 (9)	C10—C11	1.3817 (19)
O4—C13	1.2576 (16)	C10—H10	0.9500
O5—C19	1.3730 (16)	C12—H12A	0.9800
O5—C22	1.4373 (18)	C12—H12B	0.9800
O6—C20	1.3696 (16)	C12—H12C	0.9800
O6—C22	1.4365 (16)	C13—C14	1.4705 (18)
C1—C2	1.5174 (18)	C14—C15	1.3382 (19)
C1—C4	1.5197 (18)	C14—H14	0.9500
C1—C3	1.5214 (19)	C15—C16	1.4597 (17)
C2—H2A	0.9800	C15—H15	0.9500
C2—H2B	0.9800	C16—C17	1.3952 (19)
C2—H2C	0.9800	C16—C21	1.4143 (19)
C3—H3A	0.9800	C17—C18	1.4025 (19)
C3—H3B	0.9800	C17—H17	0.9500
C3—H3C	0.9800	C18—C19	1.369 (2)
C4—C5	1.5209 (18)	C18—H18	0.9500
C4—H4A	0.9900	C19—C20	1.3893 (19)
C4—H4B	0.9900	C20—C21	1.3662 (18)
C5—C6	1.5125 (18)	C21—H21	0.9500
C5—H5A	0.9900	C22—H22A	0.9900
C5—H5B	0.9900	C22—H22B	0.9900
C6—C7	1.3854 (18)		
			
C7—O1—C1	117.71 (10)	C10—C9—C8	121.89 (12)
C11—O2—C12	117.22 (11)	C11—C10—C9	119.26 (12)
C9—O3—H3	102.1 (14)	C11—C10—H10	120.4
C19—O5—C22	106.19 (10)	C9—C10—H10	120.4
C20—O6—C22	106.22 (10)	O2—C11—C10	123.78 (12)
O1—C1—C2	104.22 (10)	O2—C11—C6	114.34 (12)
O1—C1—C4	108.14 (10)	C10—C11—C6	121.87 (12)
C2—C1—C4	111.23 (11)	O2—C12—H12A	109.5
O1—C1—C3	108.13 (10)	O2—C12—H12B	109.5
C2—C1—C3	111.03 (11)	H12A—C12—H12B	109.5
C4—C1—C3	113.57 (11)	O2—C12—H12C	109.5
C1—C2—H2A	109.5	H12A—C12—H12C	109.5
C1—C2—H2B	109.5	H12B—C12—H12C	109.5
H2A—C2—H2B	109.5	O4—C13—C8	119.09 (11)
C1—C2—H2C	109.5	O4—C13—C14	117.30 (11)
H2A—C2—H2C	109.5	C8—C13—C14	123.58 (12)
H2B—C2—H2C	109.5	C15—C14—C13	120.33 (12)
C1—C3—H3A	109.5	C15—C14—H14	119.8
C1—C3—H3B	109.5	C13—C14—H14	119.8
H3A—C3—H3B	109.5	C14—C15—C16	126.31 (13)
C1—C3—H3C	109.5	C14—C15—H15	116.8
H3A—C3—H3C	109.5	C16—C15—H15	116.8
H3B—C3—H3C	109.5	C17—C16—C21	119.31 (12)
C1—C4—C5	110.62 (10)	C17—C16—C15	118.98 (12)
C1—C4—H4A	109.5	C21—C16—C15	121.71 (12)
C5—C4—H4A	109.5	C16—C17—C18	122.70 (13)
C1—C4—H4B	109.5	C16—C17—H17	118.7
C5—C4—H4B	109.5	C18—C17—H17	118.7
H4A—C4—H4B	108.1	C19—C18—C17	116.28 (13)
C6—C5—C4	110.81 (11)	C19—C18—H18	121.9
C6—C5—H5A	109.5	C17—C18—H18	121.9
C4—C5—H5A	109.5	C18—C19—O5	128.42 (13)
C6—C5—H5B	109.5	C18—C19—C20	121.91 (12)
C4—C5—H5B	109.5	O5—C19—C20	109.67 (12)
H5A—C5—H5B	108.1	C21—C20—O6	127.62 (12)
C7—C6—C11	118.26 (12)	C21—C20—C19	122.44 (13)
C7—C6—C5	122.07 (11)	O6—C20—C19	109.94 (11)
C11—C6—C5	119.64 (11)	C20—C21—C16	117.36 (12)
O1—C7—C6	121.27 (12)	C20—C21—H21	121.3
O1—C7—C8	116.29 (11)	C16—C21—H21	121.3
C6—C7—C8	122.42 (12)	O5—C22—O6	107.56 (10)
C9—C8—C7	116.06 (11)	O5—C22—H22A	110.2
C9—C8—C13	117.93 (11)	O6—C22—H22A	110.2
C7—C8—C13	126.00 (11)	O5—C22—H22B	110.2
O3—C9—C10	116.51 (11)	O6—C22—H22B	110.2
O3—C9—C8	121.58 (12)	H22A—C22—H22B	108.5
			
C7—O1—C1—C2	168.34 (10)	C7—C6—C11—C10	4.07 (19)
C7—O1—C1—C4	49.90 (13)	C5—C6—C11—C10	−174.18 (12)
C7—O1—C1—C3	−73.46 (13)	C9—C8—C13—O4	−4.41 (18)
O1—C1—C4—C5	−61.88 (13)	C7—C8—C13—O4	176.85 (11)
C2—C1—C4—C5	−175.75 (11)	C9—C8—C13—C14	173.88 (11)
C3—C1—C4—C5	58.13 (15)	C7—C8—C13—C14	−4.9 (2)
C1—C4—C5—C6	42.19 (14)	O4—C13—C14—C15	−11.16 (19)
C4—C5—C6—C7	−10.10 (17)	C8—C13—C14—C15	170.52 (12)
C4—C5—C6—C11	168.08 (11)	C13—C14—C15—C16	−179.92 (11)
C1—O1—C7—C6	−17.72 (17)	C14—C15—C16—C17	178.18 (13)
C1—O1—C7—C8	163.98 (10)	C14—C15—C16—C21	−1.5 (2)
C11—C6—C7—O1	178.33 (11)	C21—C16—C17—C18	0.1 (2)
C5—C6—C7—O1	−3.47 (19)	C15—C16—C17—C18	−179.65 (12)
C11—C6—C7—C8	−3.48 (19)	C16—C17—C18—C19	−0.5 (2)
C5—C6—C7—C8	174.72 (12)	C17—C18—C19—O5	−178.80 (13)
O1—C7—C8—C9	177.73 (10)	C17—C18—C19—C20	0.6 (2)
C6—C7—C8—C9	−0.54 (18)	C22—O5—C19—C18	−176.32 (13)
O1—C7—C8—C13	−3.51 (18)	C22—O5—C19—C20	4.25 (14)
C6—C7—C8—C13	178.22 (12)	C22—O6—C20—C21	176.72 (13)
C7—C8—C9—O3	−177.31 (11)	C22—O6—C20—C19	−3.77 (14)
C13—C8—C9—O3	3.82 (18)	C18—C19—C20—C21	−0.2 (2)
C7—C8—C9—C10	4.29 (18)	O5—C19—C20—C21	179.23 (12)
C13—C8—C9—C10	−174.58 (11)	C18—C19—C20—O6	−179.78 (12)
O3—C9—C10—C11	177.68 (11)	O5—C19—C20—O6	−0.31 (15)
C8—C9—C10—C11	−3.84 (19)	O6—C20—C21—C16	179.26 (12)
C12—O2—C11—C10	−4.53 (19)	C19—C20—C21—C16	−0.19 (19)
C12—O2—C11—C6	176.54 (11)	C17—C16—C21—C20	0.27 (18)
C9—C10—C11—O2	−179.33 (12)	C15—C16—C21—C20	179.99 (12)
C9—C10—C11—C6	−0.49 (19)	C19—O5—C22—O6	−6.49 (14)
C7—C6—C11—O2	−176.98 (11)	C20—O6—C22—O5	6.31 (13)
C5—C6—C11—O2	4.77 (17)		

### Hydrogen-bond geometry (Å, °)

**Table:**

*D*—H···*A*	*D*—H	H···*A*	*D*···*A*	*D*—H···*A*
O3—H3···O4	0.86 (1)	1.63 (1)	2.453 (1)	158 (2)
